# The Administrative Problem of Workhouse Nursing

**Published:** 1914-08-08

**Authors:** 


### THE ADMINISTRATIVE PROBLEM OF WORKHOUSE NURSING.

From time to time we have drawn attention in these columns to the defects of the provisions of the Poor-Law Institutions Order as regards the nursing of the sick in non-separated workhouse infirmaries and sick wards. Within the last few days an important deputation from the Workhouse Nursing Association, which included amongst its members Lord Henry Bentinck, Miss Haldane, and Mr. W. H. Dickinson, was received by Mr. Herbert Lewis, the Parliamentary Secretary of the Local Government Board, on behalf of the President, Mr. Herbert Samuel. The chief points brought before the Board by the Association were: (1) That while nursing by inmates is prohibited, the Order does not forbid the employment of an inmate for certain duties which materially affect the comfort of the sick, such as bed-making and the washing of helpless cases. (2) That the authority given the master and matron over the superintendent or head nurse leads to serious friction, and renders her position an almost impossible one. (3) That the medical officer, and not the workhouse master, should be empowered to engage temporary nurses. (4) That a most serious omission in the new Order is the absence of any provision whereby the superintendent or head nurse can communicate direct with the medical officer. (5) That no provision is made for the proper classification of sick children, who are now quite usually, even in large infirmaries, located in adult wards, an arrangement to be deprecated on many grounds.

Although we are in general agreement with these criticisms, it is only fair to the Local Government Board to point out that in some instances they need modification. For example, as regards the first point raised, the matter is very largely in the hands of the medical officer. The Order distinctly states that "An inmate shall not, unless approved for the particular employment by the medical officer and acting under the immediate supervision of a paid officer, be employed in *any capacity* in the sick wards." If this provision is strictly carried out no hardship is likely to arise, for one cannot believe a medical man would sanction the employment of an inmate for carrying out in connection with sick patients duties which require skilled attendants for their proper performance. Again, with regard to point (4), the Order does make provision for direct communication between the superintendent nurse and the medical officer as regards certain important matters. She is directed to inform the medical officer of any defects observed in the arrangement of the sick wards and of any matters requiring consideration in connection therewith or with the nursing of the sick. Where the Order does fail in this respect lies in the fact that it makes it the duty of the superintendent nurse to report to the master (and not to the medical officer) any negligence or other misconduct on the part of any female officer or servant in the sick wards, and any case in which restraint or compulsion may have been used towards any female lunatic or alleged lunatic.

But although we criticise the points raised by the deputation, we do so only because we do not wish the case it presented to be weakened by statements which will not bear investigation. On the main point—the impossible position of the superintendent nurse under the new Order—we entirely agree with the deputation. We agree, too, with their contention that every Poor-Law institution in which there are any sick inmates should include at least one fully trained and certificated nurse, and that the Local Government Board ought to establish a nursing service under the control of the department, so as to attract women to Poor-Law nursing who now look upon it as inferior to hospital and private nursing. These three points in the programme of the Association are very closely connected. Until the superintendent nurse is freed entirely from the control of the workhouse master well-trained nurses will avoid—and rightly avoid—applying for posts in a non-separated workhouse infirmary.

